# 

**Published:** 1886-08

**Authors:** 


Page

ORIGINAL COMMUNICATIONS:

Bacillus Tuberculosis. Conclusions. H. D. Schmidt ... 97
Hydrophobia and Imagination. W. H. Forwood ... 125
Intubation of the Larynx. F. E. Waxham ... 132

EDITORIAL:

Impulsive and Partial Insanity ... 144

SOCIETY REPORTS:

CHICAGO GYNÆCOLOGICAL SOCIETY. *Meeting May 28.*

Abdominal Section for Pelvic Abscess ... 146
A Case of Supernumerary Digits. D. T. Nelson ... 152
Uterine Fibroids and Ergot. C. T. Parkes ... 153
Occlusion of the Os Uteri an Impediment to Labour. F. E. Waxham ... 160

CHICAGO MEDICAL SOCIETY. *Meeting June 7.*

The Intra-Uterine Steam in Flexions. A. R. Jackson ... 163
A Case of Pistol-Shot Wound. A. R. Small ... 179
The Danger in Specialism. A. S. Houghton ... 181

DOMESTIC CORRESPONDENCE:

Hot-Water Baths in Puerperal Peritonitis. F. W. Fitz-Gerald ... 183

ABSTRACT:

Recent Progress in Dermatology. H. J. Reynolds ... 184

BOOK REVIEWS:

Student's Manual of Venereal Diseases. B. Hill and A. Cooper ... 188
Manual of Diseases of the Skin ... 188
Cutaneous Memoranda. H. G. Piffard ... 189
Venereal Memoranda. P. A. Morrow ... 189
Comparative Anatomy and Physiology. F. J. Bell ... 190
Practical Anatomy. C. Heath ... 190

BOOKS RECEIVED ... 191

PAMPHLETS RECEIVED ... 192

